# Growth of *Oncorhynchus mykiss* (Rainbow Trout) through a Recirculation System in the Foothills of the Extreme North of Chile

**DOI:** 10.3390/ani14172567

**Published:** 2024-09-03

**Authors:** Renzo Pepe-Victoriano, Piera Pepe-Vargas, Moira Yañez-Valenzuela, Héctor Aravena-Ambrosetti, Germán Olivares-Cantillano, Felipe Méndez-Abarca, Jordan I. Huanacuni, Sheda Méndez, Luis Espinoza-Ramos

**Affiliations:** 1 Área de Biología Marina y Acuicultura, Facultad de Recursos Naturales Renovables, Universidad Arturo Prat, Arica 1000000, Chile; moiraconstanza1720@gmail.com (M.Y.-V.); hector.aravena.ambrosetti@gmail.com (H.A.-A.); f.mendez.abarca@gmail.com (F.M.-A.); jordan.92ihp@gmail.com (J.I.H.); smendez@estudiantesunap.cl (S.M.); 2 Núcleo de Investigación Aplicada e Innovación en Ciencias Biológicas, Facultad de Recursos Naturales Renovables, Universidad Arturo Prat, Iquique 1110939, Chile; 3 Facultad de Ciencias Naturales y Oceanográficas, Universidad de Concepción, Concepción 4030000, Chile; piera.pepe.vargas@gmail.com; 4 Programa de Magíster en Acuicultura, Mención en Cultivos de Recursos Hidrobiológicos y Mención en Acuaponia, Facultad de Recursos Naturales Renovables, Universidad Arturo Prat, Arica 1031597, Chile; 5 Piscicultura Río Blanco, Federico Albert Taupp, Pontificia Universidad Católica de Valparaíso, Valparaíso 2340000, Chile; german.olivares@pucv.cl; 6 Finfish Aquaculture Sociedad Anónima Cerrada, Tacna 23004, Peru; 7 Departamento de Ingenieria Pesquera, Facultad de Ingenieria y Arquitectura, Universidad Nacional de Moquegua (UNAM), Ilo 18601, Peru; 8 Escuela de Ingeniería Pesquera, Universidad Nacional Jorge Basadre Grohmann, Tacna 23004, Peru; lespinozar@unjbg.edu.pe

**Keywords:** aquaculture, pre-cordilleran communities, photovoltaic system, salmonid, trout transport

## Abstract

**Simple Summary:**

Aquaculture is presented as a viable solution to overfishing and the growing demand for marine products. This study investigates the rearing of rainbow trout (*Oncorhynchus mykiss*) in a recirculating system at 3000 m above sea level, in Copaquilla, northern Chile. A total of 5000 juvenile trout were transported from the Rio Blanco fish farm over 2100 km. For 20 months, growth parameters were evaluated, including specific growth rate, weight gain, feed conversion, survival, and Fulton’s condition factor. The results indicated normal growth and good quality, confirming the feasibility of trout aquaculture at a high altitude. This suggests new opportunities for aquaculture in the Andean region of northern Chile.

**Abstract:**

Given the overexploitation of fisheries and the growing consumption of sea products, aquaculture is emerging as an alternative to meet the demand for protein at regional, national, and global levels. In northern Chile, the foothills of the Andes offer an opportunity for sustainable economic diversification. In this study, results of a rainbow trout (*Oncorhynchus mykiss*) culture in a recirculation system are presented, analyzing its growth and performance under altitude conditions. The research was carried out in Copaquilla, a small area in the foothills of northern Chile, 3000 m above sea level. Five thousand 15 g juvenile trout were acquired and transported by land from the Rio Blanco fish farm, successfully traveling more than 2100 km. During the 20-month-long culture, several growth parameters were evaluated, including specific growth rate, percentage of weight growth, feed conversion factor, survival, and Fulton’s condition factor. All these parameters were within the normal growth range for trout, evidencing good-quality growth of the organisms. The results offer evidence that rainbow trout farming at 3000 m above sea level is viable and presents a viable performance, opening new opportunities for aquaculture in northern Chile.

## 1. Introduction

Seafood products represent a viable alternative in response to global food consumption demand. The overexploitation of fisheries and the increase in consumption of said products have caused aquaculture to become a viable alternative to expand and contribute to providing necessary food supply as a response to the regional, national, and global protein market [1]. In this scenario, the production of salmonid fish through aquaculture, such as rainbow trout (*Oncorhynchus mykiss*), has become massive in recent years due to several reasons. Firstly, its nutritional potential, as it is a fish that contains large amounts of omega-3 fatty acids, vitamins, and essential minerals. Secondly, it represents an important source of high-quality proteins [2,3,4]. In addition, its cultivation leads to the diversification of economic opportunities in developing countries, through the generation of new jobs and the reduction in migration due to lack of income, and to the improvement of life quality in rural communities [5,6].

In Chile, aquaculture is an activity of great economic importance as the country is ranked as one of the world’s main producers of farmed trout [7]. Salmonids—non-native animals for this region—present high production values, mainly due to higher market prices [7], positioning them as the most important productive group of fish. As of 2022, there were 1000 sea farming centers registered in the Registro Nacional de Acuicultura (National Aquaculture Registry), with an average of 9 ha [7]. The main crops are concentrated in the Región de los Lagos, Aysén and Magallanes in southern Chile. The region of Arica and Parinacota, in the extreme north of Chile, is characterized as an arid area, with zero annual rainfall, and an average temperature of 18 °C [8]. Thus, this area has low aquaculture activity [9,10]. An alternative for fish farming in areas with water scarcity is the use of closed water recirculation systems (SRA) [11], whose main feature is the reuse and saving of this precious resource [12,13,14]. In addition, the high solar radiation in this area represents an opportunity for solar energy usage for different applications, such as photovoltaic technologies [15]. Considering these aspects, previous studies have named aquaculture in the region of Arica and Parinacota as an option of great productive value. In addition, thanks to the implementation of technologies that take advantage of solar energy, it is established as a sustainable and environmentally friendly economic activity [16].

There is, therefore, the possibility of implementing crops of commercially valuable freshwater species as a strategy to harness the existing agricultural infrastructure in the sector. Irrigation storage tanks, greenhouses, and hydraulic systems, among others, are readily available. The possibility of reusing water as many times as necessary, with SAR, before its being derived as a destination for irrigation of plants and vegetables is also within reach [17].

This is even more relevant if we consider that communities in the foothills of the region, such as Copaquilla, located at 3000 m above sea level, depend mainly on agricultural, livestock, and, to a lesser extent, tourism activities. The latter has suffered deterioration and abandonment due to the lack of diversifying alternatives that would prevent population migration; this is one of the main dilemmas of the territory. This scenario reinforces aquaculture as an alternative for the diversification of economic activities in the region, offering a viable and sustainable alternative to promote local development.

One limitation in the development of aquaculture activities in this sector is obtaining eggs and/or juveniles of species for culture. The transport of fish in aquaculture is a practice that often becomes necessary and critical within the production chain, but it is an operation that requires a great deal of care. Although technologies that allow diversifying the forms of transport have been generated, the transport of live fish is an activity that directly triggers stress in the fish, which compromises their welfare and survival [18,19].

Providing suitable conditions is critical for trout culture, especially at high altitudes where there is a significant decrease in the dissolved oxygen content of water. Water temperature also plays a crucial role in the ability to hold dissolved oxygen; temperatures that are too high can reduce the amount of oxygen that can be dissolved. Therefore, it is necessary to take appropriate measures to ensure an optimal environment that promotes the healthy growth and development of rainbow trout.

During the culture of any fish, it is necessary to monitor and record its growth. The implementation of growth curves, obtained from weight records of the cultured organisms over time, allows us to observe their development as they increase in weight. In addition, aquaculturists consider a series of parameters such as specific growth rate (SGR), percentage growth in weight (%IP), and feed conversion factor (FCA), among others. Although trout farming is very common, the growth of organisms can vary significantly between experiences due to the specific conditions of the culture and the attention dedicated to its management [20]. Growth parameter registration and analysis during a culture will allow us to have control over the increase in weight of the organisms and, thus, make appropriate decisions regarding the culture conditions.

This study presents the results of the culture of rainbow trout (*Oncorhynchus mykiss*) in a recirculation system located in the foothills of northern Chile, at an altitude of 3000 m above sea level. The culture was carried out using juveniles that were transported from a fish farm in the city of Los Andes, traveling more than 2000 km. The performance of the trout was analyzed in terms of growth and water quality, allowing the identification of key factors in their optimal development in higher altitude environments.

## 2. Materials and Methods

### 2.1. Study Area

**Figure 1:**
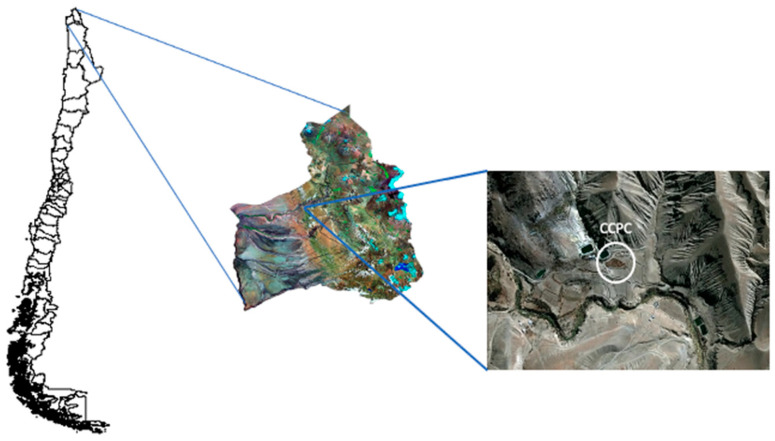
Geographical location of the Pukara de Copaquilla Cultivation Center (CCPC).

The present investigation was carried out in the foothills of northern Chile, in a town called Copaquilla (18°23′43″ S, 69°37′58″ W), 90 km inland from Arica city, at 3000 m asl (Figure 1).

### 2.2. Characteristics of Trout

**Figure 2:**
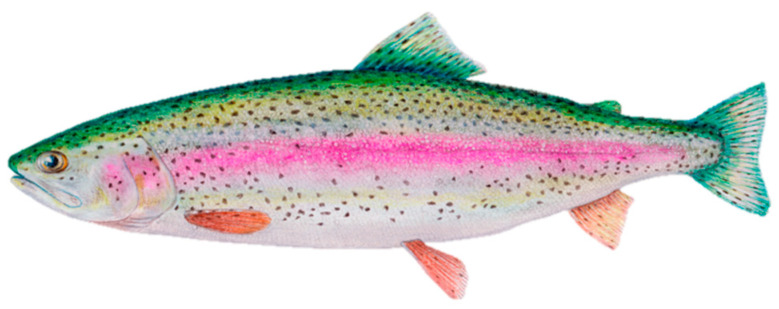
Adult specimen of *Oncorhynchus mykiss* (rainbow trout). Our own image.

Rainbow trout (Figure 2) is a typical species of inland waters, living in lotic and lentic environments. Part of the salmonid family, of the Oncorhynchus genus, it is the most widely used fish for captive culture. In Chile, the species *Oncorhynchus mykiss*, or rainbow trout, was introduced in 1905 and is currently one of the most widely cultivated salmonid species, due to its resistance and ease of breeding [21,22].

The temperature tolerance range for rainbow trout varies from 0 to 25 °C [23], with an optimum of 9 to 17 °C for good culture development [24]. However, to promote optimal growth, a slightly higher temperature range of 15 to 20 °C is preferred [25].

Morphologically, the rainbow trout has an elongated and fusiform body, adapted for fast and agile swimming. Although its coloration may vary according to its environment and degree of stress, it commonly presents olive tones on the back, silver on the sides and a pattern of black dots on the head, body, and caudal fin, often exhibiting a pink or reddish stripe along the sides that resembles a rainbow [26].

### 2.3. Collection of Biological Material and Transportation

**Figure 3:**
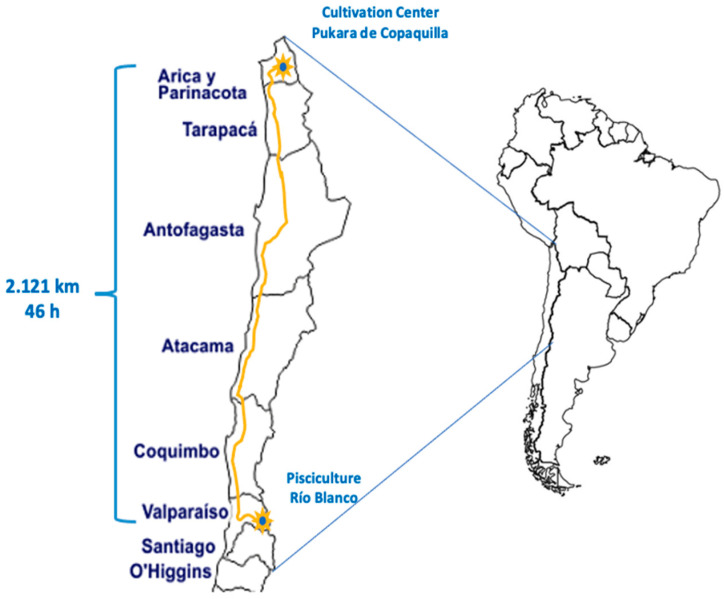
Trout transport route.

Five thousand juvenile trout weighing approximately 15 g were acquired at Rio Blanco Federico Albert Taupp fish farm, managed by the Pontificia Universidad Católica de Valparaíso, located in the city of Los Andes, Valparaíso Region. The juveniles were transferred to the study area in Copaquilla, Arica and Parinacota Region (Figure 3).

A transport truck and a support truck were used. These were disinfected before entering the Rio Blanco fish farm premises, following Chilean fish transport procedure regulations (Exempt Resolution 2010, of the Ministry of Economy, Development and Tourism).

The truck was equipped with 8 thermoplastic tanks of 1000 L, two oxygen diffusers in each tank and 6 oxygen tubes with a capacity of 9 m^3^. Temperature regulation was achieved through an incorporated thermos in the truck; due to technical complexity, it was impossible to maintain temperature control in the tanks themselves.

In the premises, the 8 tanks were filled with water at 7 °C and loaded at a rate of 625 fish per tank. At the end of the loading procedure, temperature and oxygen data were obtained.

During the 42 h of the trip, a structured sampling approach was implemented with a temperature and oxygen recording system, measured with a portable oxygenometer (YSI, Model i55, Hanna, Santiago, Chile), and a general observation was performed of the organisms, looking for unusual behavioral activity such as abnormal swimming activity, altered opercular rhythm, or any other indication of stress. During the first 6 h, hourly measurements and observations were made. Then, during the following twelve hours, measurements and observations were taken every two hours. Subsequently, during the next twelve hours, data collection continued every three hours. Finally, in the last hours of the trip, parameters were recorded every five hours. This strategic sampling plan allowed us to capture a detailed and complete picture of tank conditions throughout the entire trip.

Between 12 and 15 h before arriving at the destination, water parameters of the tanks were regulated, increasing temperature and decreasing oxygen, in order to adapt the trout to the conditions of the Copaquilla culture.

Once the fish arrived at the top of Copaquilla, dead specimens were discarded and those that survived were deposited in a tank containing 800 L of water on a different truck. This was the only way to carry them to the destination as logistical restrictions on the road prevented direct access to the culture area, located 5 km from the sector where the truck was located. Eight trips were made to vacate the eight tanks. The fish in the culture center were distributed in equal quantities in 4 tanks of 40 m^3^ (1250 fish per tank).

### 2.4. Recirculation System

**Figure 4:**
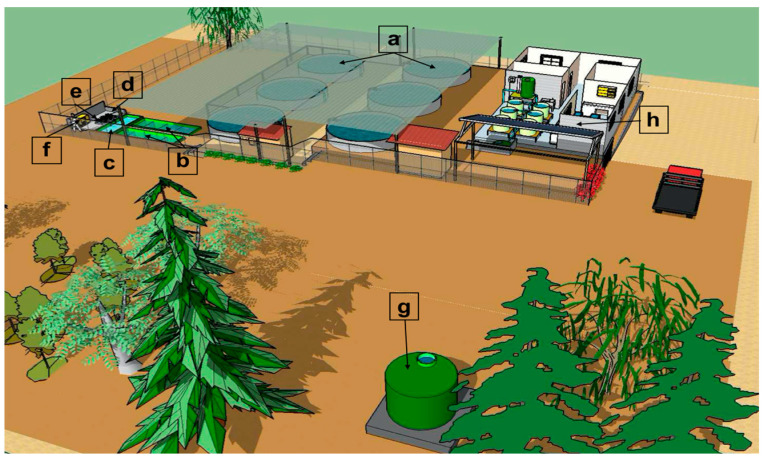
Schematic diagram of the recirculation system installed at the Copaquilla Pukara Cultivation Center. (**a**) Cultivation tanks; (**b**) decanting tanks; (**c**) biofilter tanks; (**d**) pumps; (**e**) blower; (**f**) oxygen generator; (**g**) header tank; (**h**) photovoltaic panels.

A closed water recirculation system was implemented (Figure 4), which consisted of six circular Australian-type tanks (Figure 4a) designed for intensive trout production. Only four were used in this experiment. This system had a central drainage system, hydraulic connections for water supply and aeration, as well as fiberglass tanks located below ground level, including two sedimentation tanks (Figure 4b) and one tank equipped with biofilters (Figure 4c). In addition, two water suction pumps (Reggio, model SM 150, Pentair, Puerto Montt, Chile) (Figure 4d), a high-pressure blower (Sweetwater, 1.5 hp) (Figure 4e), and an oxygen generator (Oxiti, 8 LPM, Pentair, Puerto Montt, Chile) (Figure 4f) were implemented.

The Australian-type intensive trout production tanks consist of corrugated galvanized steel and have a diameter of 5.4 m and a nominal height of 1.76 m, with a maximum water capacity of 40 m^3^. Water was supplied to each tank through a central distribution pipe and lateral connections that stack each cultivation unit. Water flow was regulated by means of PVC valves strategically arranged to generate a circular water flow.

Internally, each tank was lined with a black non-toxic geomembrane with a thickness of 0.7 mm, acting as a waterproofing layer. In the center of each tank was a drain with a 90 mm diameter outlet and a drainage system with 5 mm diameter openings.

Additionally, an air distribution pipeline was installed, which, through a blower, provided continuous aeration and oxygenation of the water column to maintain optimal dissolved oxygen levels.

Additionally, a piping network was implemented from an oxygen generator extending to all the tanks, which readily supplied oxygen in case of emergency. To guarantee the continuity of the oxygen supply, a cylinder connected to the same network was added, ensuring supply in case of generator failure.

The water evacuated from the six tanks flowed through a PVC conduit located below ground level to the sedimentation tanks. These internally divided tanks facilitated the sedimentation of suspended solids.

Accumulated residues at the bottom of the sedimentation tanks were periodically removed by means of a submersible pump. On a weekly basis, fresh water was added to these tanks, considering between 0.5 and 1% of the total volume of the system, with the objective of maintaining the volume of the culture and eliminating by-products of the nitrification process, such as nitrates. This procedure compensated for evaporation and system management losses.

Subsequently, water from the settling tanks was fed into a fluidized submerged biofilter, which contained plastic parts of construction with an ample surface area for the attachment of nitrifying bacteria, called “biofilter substrates”. The nitrifying bacteria convert the ammonia nitrogen released by fish into nitrite and then into nitrate—a molecule less harmful to aquatic organisms. The nitrification process requires the presence of dissolved oxygen; therefore, the volume of air generated by the aerator was used in the system. Diffusion hoses were strategically arranged to ensure uniform movement of biofilter substrates. In this way, an efficient distribution of ammonia, nitrogen, and oxygen over the entire surface of the biofilter was achieved, avoiding the accumulation of solids.

The treated water in this tank was returned to the headwater tank (Figure 4g), with a volume of 10 m^3^, where it underwent a gravity cascade aeration process before being directed to the production tanks. This additional step ensured that water fed into production tanks maintained adequate oxygen levels, thus contributing to an optimal trout-rearing environment.

### 2.5. Photovoltaic System (PS)

A photovoltaic system (PS) was implemented (Figure 4h), which consisted of 16 panels of 250 W, a Studer XTH 8000 inverter, a Track VT-80 controller, 12 batteries of 2 V OPzS solar power type of 2900 Ah, plus a few accessories that made up the PV system.

This PS played a crucial role in providing the essential electrical power for the efficient functioning and operation of the various equipment that made up the integrated aquaculture recirculation system. As an additional safety measure, a generator set was incorporated into the PS to act as a backup in emergency situations, thus ensuring operational continuity in any unforeseen circumstances. This integrated approach allowed for sustainable and autonomous management of the infrastructure, optimizing the efficiency and reliability of the system.

### 2.6. Water Quality

Water quality monitoring was carried out in the four culture tanks where fish were kept. This monitoring was carried out during the entire culture period and consisted of temperature and oxygen measurements performed daily, three times a day (at 08:00, 13:00, and 18:00 h) (YSI portable oxygenometer, model I55, Hanna, Santiago, Chile). Ammonium and nitrate measurements were taken every 15 days (Hanna Multiparameter Photometer, model HI83303, Hanna, Santiago, Chile) to avoid high nitrate concentrations in the culture water. Water renewal was implemented in the system, equivalent to 40% of the total volume, whenever nitrate measurement values were high. Systematic monitoring of water parameters guaranteed optimal conditions for trout development, ensuring an adequate and controlled aquatic environment.

### 2.7. Growth

For each tank, sampling was carried out every 15 days, so that growth of the organisms was regularly evaluated and documented. In each sampling session, a representative sample was selected, which consisted of 2% to 3% of the tank population. The selected trout were temporarily placed in a container for subsequent individual weighing using a digital scale (Mocco model V-1026). During this process, no chemical agent was used to sedate the fish, and diligence was exercised to minimize stress and prevent any possible damage to the specimens. Once sampling was completed, specimens were returned to their tank.

The fish were fed three times a day: in the morning, at noon, and in the afternoon. Feed rations were calculated considering 2% of the biomass present in each tank, which was calculated through sampling. The feed used for growth was Power 250. Ast.100 ppm with 40% protein.

The determination of growth variables was carried out considering five parameters: specific growth rate (SGR), percentage of growth in weight (%IP), feed conversion ratio (FCR), percentage of survival (%S), and Fulton’s condition factor (K). The formulas and calculations performed for each of these parameters are explained below:

(a) The specific growth rate (SGR), which corresponds to the measure of the percentage of growth in body weight per day [27], was calculated as follows:
  SGR = ((ln wf − ln wi)/t) ∗ 100(1)
  where wi = initial weight; wf = final weight; t = time in days fed.
(b) The percentage of growth in weight (%IP), estimated through the difference between final biomass minus initial biomass times 100 [27], was calculated as follows:
  % IP = [[wf − wi]/wi] ∗ 100(2)
  where wi = initial weight; wf = final weight.
(c) The feed conversion ratio (FCR) is an indicator that expresses weight gain of a cultured organism in relation to the weight of feed used. It was calculated by the formula:
  FCR = ac/ip(3)
  where ac = food consumed (Kg); ip = increase in weight (Kg).
(d) Survival was determined through the analysis of daily mortalities per tank, which were counted at the end of the experiment, obtaining the real number of live fish. This is expressed as survival percentage (%S) [27], via the following expression:
  % S = [nf/ni] ∗ 100(4)
  where ni = initial number of individuals; nf = final number of individuals.
(e) Fulton’s condition factor (K) is estimated to determine the degree of well-being or robustness of trout in two stages: fry and juvenile. This factor is used to determine the length (cm) of the fish according to their weight (g) or, conversely, to determine the weight of the fish.
  K = 100 ∗ (W/L^3^) (5)
  where W = wet body weight in grams; L = length in centimeters.

### 2.8. Statistical Analysis

Water quality and growth parameter data for each tank were compared using the Shapiro–Wilk normality test and Bartlett’s test to check the homogeneity of variance. If statistical assumptions were not met, data were evaluated with non-parametric statistics using the Kruskal–Wallis test. Since there was no significant difference between tanks, the results of the analyses are presented as an average of all obtained values.

## 3. Results

### 3.1. Trout Transport

**Figure 5:**
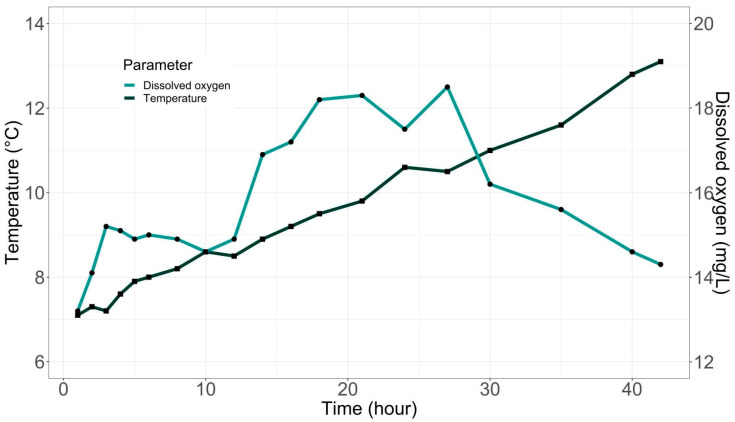
Evolution of temperature and oxygen parameters during trout transport from the Rio Blanco, Federico Albert Taupp Fish Farm, Los Andes (Valparaíso Region) to Copaquilla (Arica and Parinacota Region).

Figure 5 shows the evolution of temperature and oxygen parameters during the 42 h of transport of organisms from the Rio Blanco fish farm, Federico Albert Taupp, in the Andes, Valparaiso Region, to the top of Copaquilla, in the Arica and Parinacota Region. It can be clearly observed that 27 h after the start of the trip, there is a decrease in dissolved oxygen in tank water attributable to the adaptation of organisms as described in the methodology section.

The temperature increased throughout the trip (Figure 5), reaching numbers close to 14 °C at the end of transportation, which was like the temperature of water in the tanks at the Copaquilla culture center.

During the transfer, 3 of the 5000 specimens transported died, attributable to crushing caused by the tank’s diffusers. Once the fish were placed in the tanks of the Copaquilla culture center, no massive mortalities were observed during the stable period (acclimatization), which allows us to rule out any type of negative effect due to thermal shock at the time of reception, as well as to dismiss any future complications associated with this phenomenon.

### 3.2. Water Quality

**Figure 6:**
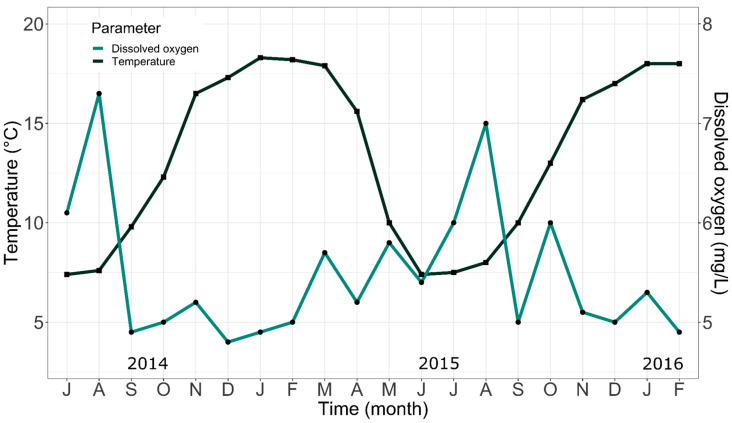
Monthly changes in temperature and oxygen levels recorded in culture tanks throughout the study.

The results of oxygen and temperature monitoring are presented as monthly averages in Figure 6. During cold months, from April to October, there was a decrease in temperature values, with records below 8 °C. In contrast, in warmer months, from November to March, there was an increase in temperature, reaching values above 18 °C between January and March. The concentration of dissolved oxygen in tank water showed an inversely proportional trend with respect to temperature, which was also influenced by seasonal changes. During cold periods, a higher level of dissolved oxygen is observed in comparison to warmer months. During high-temperature periods, increases in oxygen consumption are expected, resulting in lower oxygen levels in water.

**Figure 7:**
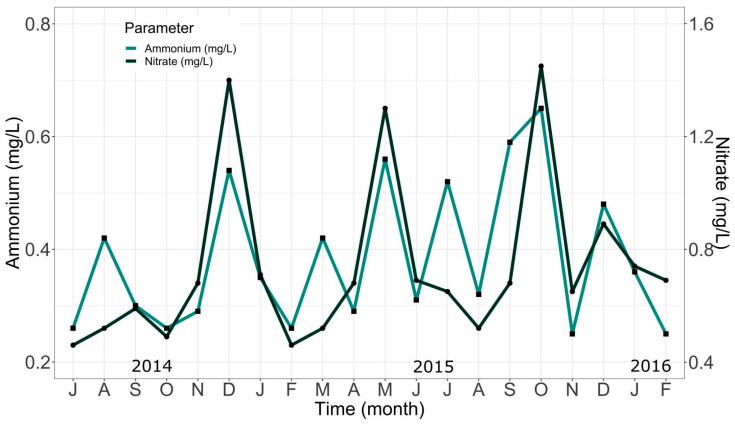
Average monthly changes in ammonium and nitrate levels recorded during the study.

On the one hand, Figure 7 shows the data for ammonium and nitrate levels during the growing season. Water renewals made in the system in response to elevated nitrate levels can be seen at the points where the curve of this parameter decreases. On the other hand, the oscillations in the ammonium values—regulated by bacteria present in the biofilter—were maintained throughout the whole experience, fluctuating between 0.25 and 0.65 mg/L. Such oscillation was also influenced by water renewals made to the system, verifiable by comparing the points of decrease where both curves coincide. Although some variations were observed, the reached values did not represent a risk for fish in the culture.

### 3.3. Growth

**Figure 8:**
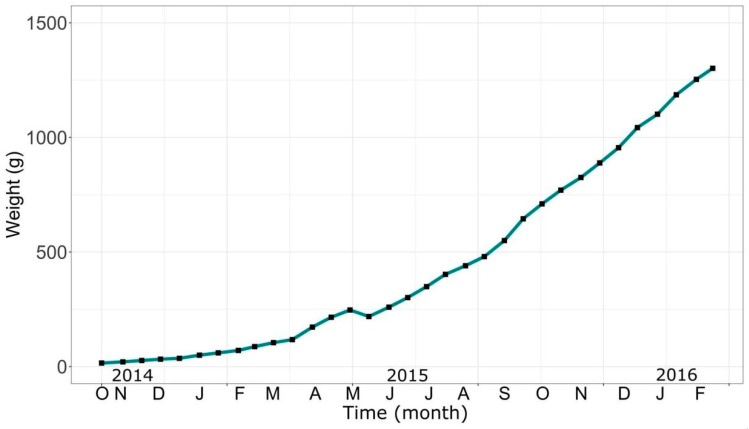
Average weight of fish from the culture tanks during the research period.

Figure 8 shows the average weight of fish cultured in the four tanks. After seven months of culture, the largest fish from each tank were selected to be conditioned in parallel as possible broodstock, which is the reason why the curve exhibits constant growth in the tanks, except for samplings of the seventh month of culture (May 2015), where a drop in growth is observed, attributable to this very extraction. The curve shows that organisms continued to grow, reaching an average weight of more than 1300 g at the end of the research—after 16 months of culture.

To facilitate the reading of data, we decided to divide the cultured organisms’ growth into two stages: the first stage we call “pre-fattening”, corresponding to the first 7 months of culture from their arrival, with an average of 15 g until organisms reached 218 g. The second stage was the “fattening” stage, corresponding to the remaining 9 months, during which fish grew from 218 g to approximately 1300 g. Growth parameters are presented, together with other data obtained in the sampling, in Table 1 and Table 2, corresponding to pre-fattening and fattening growth stages, respectively.

## 4. Discussion

In Chile, the salmonid aquaculture industry has been concentrated mainly in the southern part of the country, in coastal areas. However, the northern foothills and mountainous areas offer untapped potential for the culture of these species. Rainbow trout culture has been established at high altitudes in countries such as Colombia, Ecuador, Bolivia, and Peru, a common practice that is carried out between 2000 and 3000 masl, either in tanks or cages over lakes. Additionally, in mountainous regions with sierras or mountain ranges, such as in Bolivia or Peru, this type of culture has been rendered successfully for several years [28,29,30,31,32]. To date, the only record maintained of a trout culture in pre-cordilleran areas of northern Chile dates between 1993 and 1995 [9]. There are no current reports of salmonid culture in this area with the level of production and technology that we present in this work.

### 4.1. Transportation

During the transfer of live fish for aquaculture, factors such as transport time, adverse environmental conditions during transport, and conditioning for the production systems to which the organisms will arrive must be considered. Neglecting these variables may eventually result in immediate mortalities and short time frames following receipt of the organisms [33].

Given the number of organisms transported in this experience and the extensive distance of more than 2000 km that this trip implied, it was considered that the most viable option for transport would be a truck equipped with tanks to contain the fish. This tank system allowed for external oxygenation, unlike other methods, such as transport in closed, airtight plastic bags with a supersaturated oxygen atmosphere, successfully used on trips of up to 24 h [34,35,36,37]. This time limit is closely related to the oxygen requirements of organisms, which, although varying from species to species, tend to increase under stress and in crowded situations, common conditions during transport [38]. Therefore, the oxygen control in the water was crucial to ensure the success of the experiment.

Along with this, another critical requirement that had to be controlled was the stocking density in the tanks, where the aim was to transport as much biomass as possible at a lower cost, without compromising the welfare of organisms [39]. Orina et al. [40] reported that the best way to transport 0.2, 0.5, and 5 g *Oreochromis niloticus* broodstock is at different densities and with transport times of up to 24 h. In their research, they conclude that smaller hatchlings (0.2 g) had higher survival, recommending higher densities (16 g/L for 0.2 g hatchlings) for transport, while larger hatchlings (5 g) had higher mortality, possibly due to the limitation of dissolved oxygen in water. In other research conducted with marine species such as *Anisotremus scapularis*, a transport density for juvenile specimens with a variation between 21.18 and 42.36 kg/m^3^ [41] was observed. Said research was carried out using polystyrene bags. Pepe-Victoriano et al. [42] point out that in transports for *Sarda chiliensis chiliensis,* the density for specimens of less than one kilogram has been recorded to be between 4 and 5 kg/m^3^. Kubitza [43] mentions that for transport, an adequate load of rainbow trout—the same species cultivated in our experience—is about 60 to 80 kg/m^3^ for fry and juveniles. Compared with the values of all mentioned investigations, our density had much lower values since the load per tank was only 9.38 kg/m^3^. Even so, it is relevant to mention that the duration of transport in the above-mentioned studies ranged between 2 and 24 h at maximum, significantly lower than the transport time of this research.

During our transport experience, water quality parameters behaved as expected. The temperature increased gradually throughout the trip, which was a predictable change, especially considering we crossed one of the driest deserts in the world, where temperatures are high, and the climate is dry. Despite this change in external conditions, temperatures remained within safe limits for trout, which can tolerate temperatures up to 25 °C [23]. As for dissolved oxygen, levels were regulated externally by allowing oxygen to enter the tanks. Despite the increase in temperature and, thus, oxygen consumption, oxygen levels in the water remained above 14 mg/L, a value that allowed for the supersaturation of this gas in the system. The temperature was also monitored to ensure that it remained within safe ranges, which correspond to values below 15 °C [44,45].

Of the 5000 fish transported, only 3 fish were registered as deceased. These deaths were attributed to crushing caused by air diffusers, which were loose inside the tank. The movement of these diffusers generated a pressure that caused the fish to impact against the walls of the container. Despite this incident, no additional deaths due to transport stress were recorded, which reinforces the positive outcome of the experience in terms of fish survival.

### 4.2. Water Quality

At 3000 m and 21 °C, the maximum concentration of dissolved oxygen in freshwater would be approximately 7.6 mg/lt. This would be well above what was recorded in the experience at temperatures below 20 degrees. Therefore, a drawback could have been a low capacity to oxygenate (aerate) the water and not the limitation of how much oxygen the water can hold at that temperature and altitude.

As mentioned above, in aquaculture, it is essential to maintain strict control and recording of water parameters to keep these values within specific ranges that favor optimal growth and development of aquatic organisms. This approach seeks to guarantee ideal environmental conditions that maximize the productivity and health of cultured species [46,47]. In the case of rainbow trout, this species, like all fish, does not have the capacity to regulate its own body temperature, which is completely determined by the aquatic environment in which it lives. Under ideal temperature conditions, these fish experience rapid growth, efficient food conversion, and greater resistance to disease, among other characteristics [48]. According to Phillips et al. [24], trout can survive in waters with temperatures ranging between 0 °C and 25 °C in their natural environment; however, for optimal growth and development in culture, the author recommends keeping temperatures in a narrower range, specifically between 9 °C and 17 °C. Other authors point out that the water temperature for trout culture should oscillate in an even smaller range, between 13 °C and 18 °C [49,50]. In our experience, temperature variations were found to be between 7 °C and 18 °C, values much closer to those suggested by Phillips et al. [24]. This high variation can be explained by the fact that our facilities were outdoors and, therefore, depended on environmental temperatures—with extreme variations typical of the pre-mountainous zone of northern Chile—and the tanks did not have any form of temperature regulation.

On the one hand, water temperature has a close relationship with the amount of dissolved oxygen, which highlights the importance of maintaining an adequate balance between these parameters. As an example, it has been reported that oxygen concentrations above 21 °C are low and not suitable for trout culture. Lack of dissolved oxygen in the water can lead to disease or even death in these organisms due to their high oxygen requirement [48]. On the other hand, high oxygen levels can cause gas embolism, a condition that can be lethal to fish [37]. Considering this relationship, seasonal variations in these factors are common and predictable: during warm months, oxygen demand by fish increases with increasing water temperature, while in cold months, the opposite is true, which is precisely what was observed in our system. Although during our entire culture experience, we did not present any problems associated with oxygen, we emphasize that our concentration ranges are different from those suggested by other authors for trout culture, such as Liñan-Giraldo [49], who suggests that oxygen parameters for the culture of this species should be in a range of 7.5 to 12 mg/L, values different from ours, which fluctuated between 4.9 mg/L and 7.4 mg/L.

In relation to nitrogen compounds, it has been reported that ammonium is the main form of nitrogen in fish metabolism [51]. In open water flow systems, these compounds are not usually a significant problem, but in recirculating systems such as the one used in this study, they are critical and limiting for trout production, as fish are especially sensitive to high levels of nitrogenous compounds [52]. The balance between ammonium produced by fish, uneaten feed, and decomposition of dead bacteria must be kept in line with the rate of ammonium removal through water renewal and biological filtration [53]. During the study, water renewals were performed and a biofilter was used to maintain this balance, and, consequently, the amount of nitrogenous compounds in the system were kept under control over time.

According to Camacho et al. [54], ammonium levels in water for trout culture should be less than 0.012 mg/L, a value significantly lower than those recorded in this experience, with values close to 0.65 mg/L. This can be explained by the fact that ammonium can exist in two forms: NH^4+^ (ionized) and NH_3_ (non-ionized). The ratio between these forms is dependent on the water pH. At a higher pH, ammonium tends to convert to NH_3_, which is more toxic to fish. Conversely, at a lower pH, ammonium remains predominantly as NH^4+^, which is less toxic. Although pH does not directly control ammonium levels, it can influence its toxicity by determining its predominant form and, thus, its availability to fish [52]. Although a permanent record of pH was not kept, measures such as water renewals or short-period feeding pauses were implemented to try to maintain a low pH. Such implementations favored the presence of ammonium in its less harmful forms. It is important to keep a continuous record of this parameter in future experiences, allowing for better control and understanding of water quality in trout culture.

### 4.3. Growth

When evaluating the growth of cultured organisms, the aquaculturist must consider multiple parameters. The quality of growth will vary according to the genetics, sex, and age of the organisms, as well as the quality of diet and environmental conditions. In our experience, different parameters widely used for growth evaluation were evaluated. The values of the FCR, corresponding to the weight gain of a cultured organism in relation to the weight of feed administered, exceeded the ideal value of this index, which corresponds to 1.0 for both pre-fattening and fattening stages [55,56]. FCR values reached 2.0 and 1.98, respectively, indicating that it was necessary to deliver twice the expected feed to obtain an increase of 1 kg of trout. This inefficiency in the usage of feed may be due to stress conditions faced by the organisms. Since the tanks were outdoors, they were exposed to weather events typical of the foothills. Noise and stressful stimuli, such as thunder or rain, did not have a significant impact on the survival of the fish in the tanks, but they could have generated stressful situations that reduced the organism’s appetite and, therefore, affected the value of this index.

Regarding the SGR, a percentage of body weight gain per day measure, the values obtained in our experience, both for pre-fattening and fattening stages, were low, compared to those obtained in similar experiences of other authors, such as Chamorro [57], with average values of 0.88 and 0.51 for pre-fattening and fattening, respectively. For juvenile trout, Chamorro [57] obtained an SGR of 2.56 to 2.91 in 90 days of culture. Although this would indicate that our organisms would have experienced a negative growth rate or a very slow growth in relation to their initial size during the period of time analyzed—due to a variety of factors, such as unfavorable environmental conditions, stress, and lack of adequate nutrients, among others—we must consider the long period of time in which the culture was carried out (17 months). Furthermore, a decrease in the SGR value as fish grow (differences between pre-fattening and fattening over time) is to be expected, considering they require more energy for their maintenance [58], which translates into a decrease in SGR.

Growth percentage (%IP) values followed a normal trend, i.e., values obtained from juveniles were higher than those obtained from adults.

Finally, we emphasize that survival values in the pre-fattening and fattening stages were high, with values of 87.2% and 93.78%, respectively. Although these are satisfactory values for culture, there is a considerable difference between both stages, which could be explained if we consider that in earlier stages of their life, such as in pre-fattening culture, fish are usually more sensitive to changes in environmental conditions, such as water quality [59]. Small variations in these conditions can have a significant impact on their health and survival—the same variations that in more adult stages would not affect them in the same way.

It is interesting to note that the Fulton’s condition factor for trout fry and juveniles is above the normal value of 1.0. This result suggests a good growth status for the fish under study. According to Estay et al. [60], values below 1.0 could indicate malnutrition or a poor general condition in the fish. Hence, the fact that our values are within what he calls “Slender Fish” further reinforces the idea of healthy growth in the examined trout.

## 5. Conclusions

Our experience has successfully demonstrated the feasibility of transporting fish over 2000 km, with virtually zero mortality. This transportation required careful planning and execution, wherein proper handling and implementation of biosecurity measures ensured not only the welfare of the fish, but also compliance with relevant regulations. This milestone marks a significant advance in the transport of live fish in Chile, providing a solid foundation for future initiatives to transport fish over long distances, contributing to the development of sustainable aquaculture in the country.

Careful control of water parameters, including temperature, dissolved oxygen, and nitrogen compounds, is critical to trout culture success. Maintaining these parameters within specific ranges is crucial to promote healthy growth and minimize the risk of disease. Careful attention to these aspects ensures ideal environmental conditions that maximize trout productivity and health in culture systems.

In addition, we highlight that the extreme north of Chile presents unique and extreme climatic conditions, with high temperatures, which represents a not sufficiently explored challenge in the field of aquaculture. This research was focused on evaluating the potential of rainbow trout (*Oncorhynchus mykiss*) culture as a suitable species for aquaculture production in this area and confirmed that its culture at an altitude of 3000 m above sea level is possible, rendering good growth performance both in the pre-fattening and fattening stages. These results also broaden our understanding of the adaptability of the species to higher-altitude environments, specifically regarding its cultivation, as a real alternative to diversify productive activities in foothill areas.

We can also conclude that our study is a contribution to the advancement of small-scale aquaculture. Often, research in this field focuses on replicating the methods used by the large salmon farming industry in Chile, producing results that are inapplicable or ineffective for small-scale aquaculture. Our work opens a new area of research, focusing on providing scientific knowledge that will boost the development of small-scale aquaculture and benefit local producers.

Finally, we conclude that our study’s results obtained for the SGR and FCR, although lower than commercial levels, reflect the controlled conditions of this pilot study, whose main objective is to evaluate commercial viability in an experimental setting. These results serve as a baseline for future improvements, which will focus on diet optimization, improved rearing conditions, implementation of genetic selection programs, and introduction of more precise feeding technologies. These strategies will allow for continued advancement in feed efficiency and growth, with the goal of reaching commercial standards in later phases of the project.

## Figures and Tables

**Table 1:** Growth parameter values for the pre-fattening stage.

Variables	Est.1	Est.2	Est.3	Est.4	Total
Food provided (kg)	524	495	515	544	2077
Initial biomass (kg)	19.54	18.50	18.60	20.60	77.30
Final biomass (kg)	281.62	266.20	276.70	292.94	1117.50
Increase in weight (g)	262.08	247.67	258.07	272.34	260.04 *
Initial density (Kg/m^3^)	0.49	0.46	0.47	0.52	0.49 *
Final density (Kg/m^3^)	7.04	6.66	6.92	7.32	6.98 *
Initial no. of fish	1250	1250	1250	1250	5000
Final no. of fish	1096	1065	1105	1094	4360
FCR	2.00	2.00	1.99	2.00	2.00
SGR	0.87	0.87	0.89	0.87	0.88
Weight gain (%)	1341.25	1336.60	1385.20	1322.00	1345.60
Survival rate (%)	87.68	85.20	88.40	87.52	87.20
Fulton’s condition factor (K)	1.80	1.80	1.80	1.70	1.70

* Values presented as an average of values obtained in the tanks.

**Table 2:** Values of growth parameters for the fattening stage.

Variables	Est.1	Est.2	Est.3	Est.4	Total
Food provided (kg)	2051.00	2090.60	2197.40	2072.20	8411.20
Initial biomass (kg)	281.62	266.20	276.70	292.94	1117.50
Final biomass (kg)	1308.94	1333.72	1401.86	1321.98	5366.50
Increase in weight (g)	1027.32	1067.52	1125.16	1029.04	1062.26 *
Initial density (Kg/m^3^)	7.04	6.66	6.92	7.32	6.99 *
Final density (Kg/m^3^)	32.72	33.34	35.05	33.05	33.54 *
Initial no. of fish	1096	1065	1105	1094	4360
Final no. of fish	1011	1019	1046	1013	4089
FCR	2.00	1.96	1.95	2.01	1.98
SGR	0.50	0.53	0.53	0.49	0.51
Weight gain (%)	364.79	401.02	406.64	351.28	380.24
Survival rate (%)	92.24	95.68	94.66	92.60	93.78
Fulton’s condition factor (K)	1.30	1.20	1.20	1.20	1.20

* Values presented as an average of values obtained in the tanks.

## Data Availability

The data presented in this study are available on request from the corresponding author. The data are not publicly available for privacy reasons.
